# LAPTM4B Allele *2 Is Associated with Breast Cancer Susceptibility and Prognosis

**DOI:** 10.1371/journal.pone.0044916

**Published:** 2012-09-12

**Authors:** Xiaoyan Li, Xiangnan Kong, Xi Chen, Ning Zhang, Liyu Jiang, Tingting Ma, Qifeng Yang

**Affiliations:** 1 Department of Breast Surgery, Qilu Hospital, Shandong University, Jinan, Shandong, People’s Republic of China; 2 Key Laboratory of Experimental Teratology, Ministry of Education and Institute of Molecular Medicine and Genetics, Shandong University School of Medicine, Jinan, Shandong, People’s Republic of China; IPO, Inst Port Oncology, Portugal

## Abstract

**Background:**

Lysosome-associated protein transmembrane 4 beta (LAPTM4B) has two alleles named LAPTM4B*1 and LAPTM4B*2. Allele *1 contains only one copy of a 19-bp sequence at the 5′UTR in the first exon, whereas this sequence of allele*2 is duplicated and arrayed as a tandem repeat. Previous studies revealed that LAPTM4B polymorphisms contribute to the risk of certain types of cancers. This study aimed to investigate the polymorphism of LAPTM4B in breast cancer by analysis the correlation of LAPTM4B genotype with breast cancer susceptibility, clinicopathologic features and prognosis.

**Methods:**

Genotyping of the LAPTM4B polymorphism was determined by PCR method. The expression levels of LAPTM4B in breast cancer tissues and breast cancer cell lines were determined by quantitative reverse-transcription PCR (qRT-PCR) analysis. The correlation of LAPTM4B genotype with clinicopathologic parameters and prognosis were assessed statistically.

**Results:**

The results of qRT-PCR analysis indicated that LAPTM4B*2 was associated with the higher level of LAPTM4B expression compared with the LAPTM4B*1 in both breast cancer cell lines and breast cancer tissues. We found that LAPTM4B*2 was associated with an increased risk for breast cancer. LAPTM4B*2 was significantly associated with higher histopathologic grade, lymph node metastasis and poor prognosis.

**Conclusion:**

LAPTM4B*2 is a risk factor associated with breast cancer susceptibility and poor prognosis. LAPTM4B*2 may be a potential predicative marker for the susceptibility, progression and metastasis of breast cancer.

## Introduction

Breast cancer is the most commonly diagnosed type of female malignancy around the world. It was reported that the expected numbers of new breast cancer cases in 2012 is 230 480, which is expected to account for 30% of all new cancer cases among women [1]. It is a heterogeneous disease which occurs based on an interaction between genetic heterogeneity and environment. It has been reported that an accumulation of genetic variants is involved in the process of breast carcinogenesis [2], [3]. Therefore, efforts to identify more molecular markers for detection and diagnosis of breast cancer are of great clinical importance.

Lysosomal-associated protein transmembrane-4 beta (LAPTM4B) was originally identified as a novel oncogene candidate in hepatocellular carcinoma. It was cloned using ﬂuorescence differential display, rapid amplification of cDNA ends and RT-PCR Subsequently, it was mapped to chromosomes 8q22 and composed of seven exons separated by six introns [4], [5]. The LAPTM4B gene has two alleles named LAPTM4B*1 and LAPTM4B*2 (GenBank numbers AY219176 and AY219177, respectively). Allele *1 contains only one copy of a 19-bp sequence at the 5′UTR in the first exon, whereas this sequence of allele *2 is duplicated and arrayed as a tandem repeat [5].

Previous findings have revealed that LAPTM4B allelic variation is associated with increased risk of cancers such as gastric cancer [6], gallbladder carcinoma [7], liver cancer [8], [9], ovarian cancer [10], cervical carcinoma [11] and colon cancer [12], but it is not associated with rectal or esophageal squamous cell carcinoma [12]. In this study, we investigated the association between LAPTM4B polymorphism and its expression in both breast cancer cell lines and tissues. We then studied the effects of LAPTM4B polymorphisms on the susceptibility of breast cancer in a case-control study. The relationship between LAPTM4B genotype and clinicopathological variables as well as prognosis of breast cancer was also analyzed statistical.

## Materials and Methods

### Cell Culture

Breast cancer cell lines MCF-7, MDA-MB-231, T47D, MDA-MB-468 were obtained from American Type Culture Collection (ATCC, Rockville, MD, USA). They were routinely cultured in appropriate medium supplemented with 10% FBS and 100 units of penicillin-streptomycin at 37°C with 5% CO_2_ in a humidified incubator.

### Patients and Samples

All the breast cancer patients involved in the study were histopathologically diagnosed at department of pathology of Qilu Hospital of Shandong University. All the diagnoses were made following the Pathology and Genetics of Tumors of Breast of World Health Organization Classification of Tumors and were made by at least two pathologists. To detect the relationship between LAPTM4B polymorphism and breast cancer susceptibility, two hundred eight breast cancer patients and two hundred eleven cancer-free healthy controls who were recruited from patients undergoing annual physical examination at Qilu Hospital of Shandong University were investigated. To analysis the association between LAPTM4B gene polymorphism and prognosis, sisty-six patients with associated clinicopathologic database and long term clinical follow up were enrolled. For all participants in this study, written informed consent was obtained as delineated by the protocol which was approved by the Ethical Committee of Shandong University.

### DNA Extraction

For both patient and control group, 1.5 ml whole blood sample was extracted from each participant and stored at −80°C. DNA from both whole blood was extracted with QIAamp DNA mini kit (Qiagen, Hilden, Germany), following the manufacturer’s instructions. DNA concentration and purity of each sample were measured by ultraviolet spectrophotometer (GE Healthcare, Piscataway, NJ, USA). DNAsamples were routinely stored at −20°C.

### Polymerase Chain Reaction (PCR) Analysis of LAPTM4B Polymorphisms

Genotyping of the LAPTM4B polymorphism was determined by PCR method. Primers were designed as follows: forward primer 5′- GCCGACTAGGGGACTGGC GGA-3′ and reverse primer 5′- CGAGAGCTCCGAGCTTCTGCC-3′. In each 25 µl reaction, 1 µl genomic DNA (100 ng/µl) was amplified by the HotStart Taq PCR MasterMix (Tiangen, Beijing, China) with 1 µl of each primer. The PCR conditions were set as follows: 94°C for 3 min, 35 cycles of 94°C for 30 s, 60°C for 30 s, and 72°C for 30 s and a final extension step of 72°C for 5 min. After electrophoresis on 3% ethidium bromide added agarose gel, photographs were taken under ultraviolet light transilluminator. To confirm our results of genotyping, several PCR products were randomly picked for sequencing.

### Quantitative Reverse-transcription PCR (qRT-PCR) Analysis

Total RNA were extracted with TRIZOL reagents according to the manufacturer’s protocol (TaKaRa, Dalian, China). Briefly, cDNA was synthesized from 1 µg of total RNA by PrimerScript RT Reagent Kit (TaKaRa). QRT-PCR was performed using a SYBR green PCR mix in Applied Biosystems StepOne and StepOnePlus Real-Time PCR Systems. The samples were loaded in quadruple, and the results of each sample were normalized to GAPDH. The experiments were repeated in triplicate to confirm the findings.

### Immunohistochemistry (IHC)

To evaluate the association between LAPTM4B gene polymorphism and clinicopathological parameters including estrogen receptor (ER), progestogen receptor (PR), and C-erbB2, tissue microarrays were made and stained by streptavidin-peroxidase-biotin (SP) immunohistochemical method as previously described [13], [14]. Evaluation of the IHC staining results was proceeded by pathologists without knowing the genotypes. The tumor was scored positive for ER and PR when ≥10% of the tumor cells on the slide irrespective of the staining intensity. C-erbB2 was evaluated according to the DAKO score; complete membranous staining observed in ≥10% of tumor cells (DAKO score 2+ and 3+) was defined as C-erbB2 positive.

### Statistical Analysis

All statistical analyses in our study were carried out with SPSS Statistics 18.0 (SPSS Inc. Chicago, Illinois, USA). Genotypic frequencies were tested for Hardy-Weinberg equilibrium using the chi-square test [15]. Differences in patient survival were determined by the Kaplan-Meier method. Multivariate analysis of prognostic factors was carried out with Cox regression analysis. P-value >0.05 was considered not deviate from the equilibrium. The statistical software package SPSS18.0 (SPSS Inc., Chicago, IL) was employed for all analysis. P values of 0.05 were defined as statistically significant.

## Results

### LAPTM4B Polymorphism and its Expression in Breast Cancer Cell Lines

**Figure 1:**
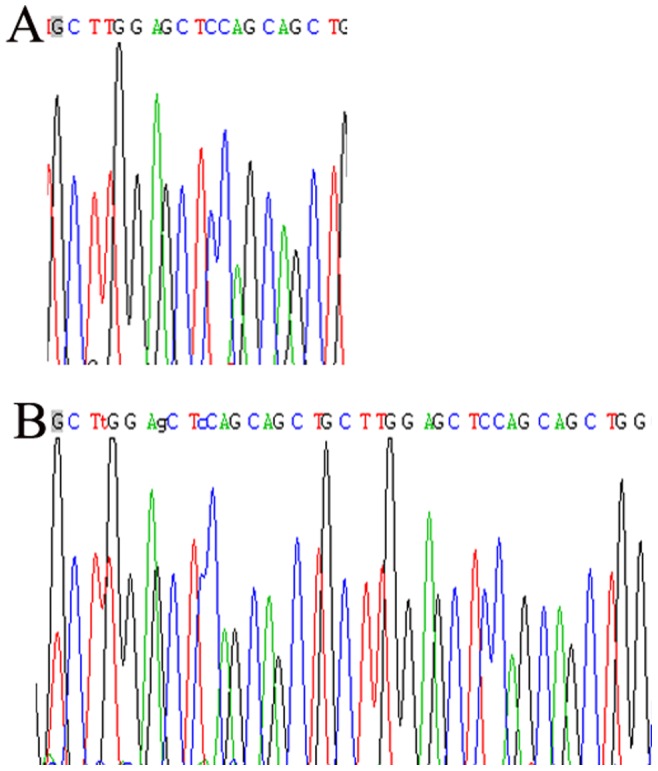
The sequencing results of PCR products. (A). LAPTM4B*1; (B).LAPTM4B*2.

**Figure 2:**
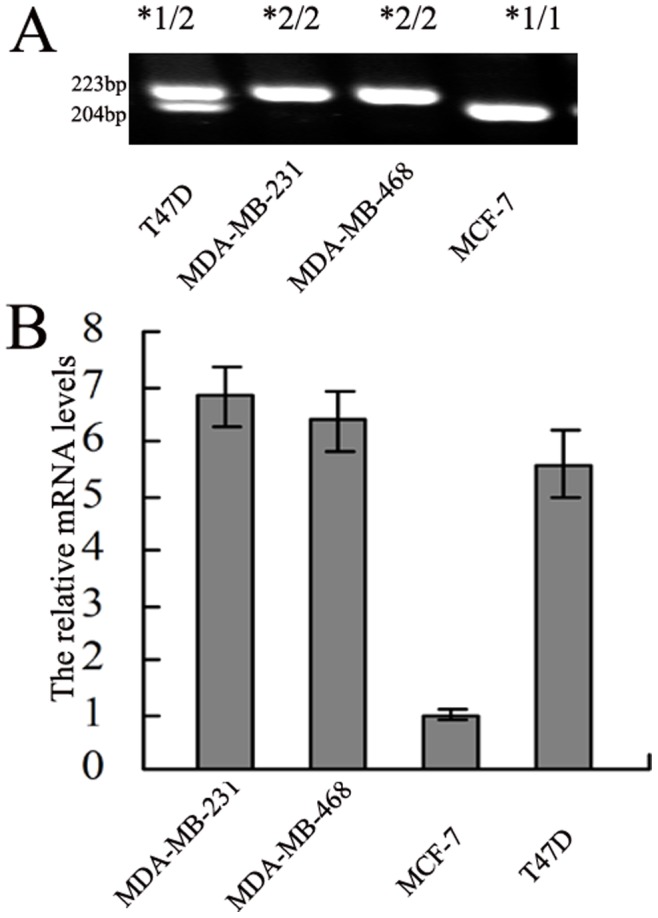
Genotype and expression of LAPTM4B protein in breast cancer cell lines. (A) The genotype of LAPTM4B in breast cancer cell lines. This polymorphism was shown by PCR using specific primers for LPTM4B. The products were analyzed by separation in a 3% agarose gel electrophoresis. (B) The mRNA levels of LAPTM4B in breast cancer cell lines. The data represent one of three independent experiments.

LAPTM4B has two alleles, so three different genotypes were generated and designated homozygous genotype LAPTM4B*1/1, homozygous genotype LAPTM4B*2/2, and heterozygous genotype LAPTM4B*1/2. Using the specific primers for LAPTM4B, we found two PCR products were amplificated with the length of 223 bp and 204 bp respectively by sequencing (Figure 1). After confirm this, we first detected the genotypes of 4 breast cancer cell lines (MCF-7, T47D, MDA-MB-231 and MDA-MB-468) were tested by PCR assay. As shown in Figure 2A, the 204 bp band codes for the homozygous genotype LAPTM4B*1/1, 223 bp band for the homozygous genotype LAPTM4B*2/2 while the heterozygous genotype LAPTM4B*1/2 has both 204 bp and 223 bp bands. We then investigated the possible linkage of LAPTM4B polymorphism with its expression level in these cell lines. Figure 2B showed the result of genotyping and mRNA levels of the 4 breast cancer cell lines. The mRNA levels of LAPTM4B/GAPDH were much higher in T47D (5.57±0.61), MDA-MB-231(6.82±0.53), MDA-MB-468 (6.36±0.57) than in MCF-7 (1.00±0.11; p = 0.0002, P<0.001, P<0.001 respectively), whereas no significant difference was found between MDA-MB-231, MDA-MB-468 and T47D (p = 0.0560, p = 0.1758 respectively). These demonstrated that a higher levels of LAPTM4B in the *2/2 genotype and LAPTM4B*1/2 genotype displayed an intermediate level, while LAPTM4B*1/1 genotype had the lower expression. Therefore, allele*2 was associated with higher level of LAPTM4B expression in breast cancer cell lines.

### LAPTM4B Polymorphism and its Expression in Breast Cancer Tissues

**Figure 3:**
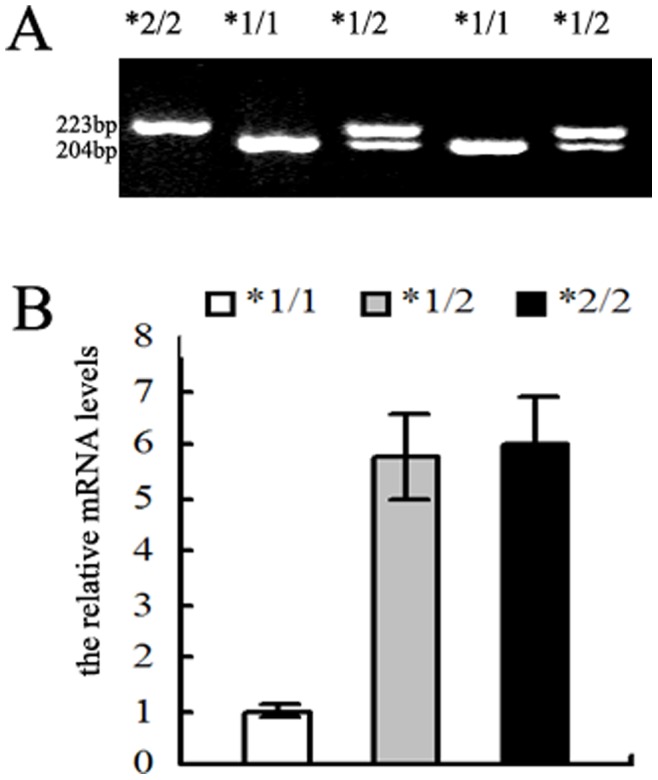
Genotype and expression of LAPTM4B protein in breast cancer tissues. (A) The genotype of LAPTM4B in representive breast cancer tissues. Lane 2, 4: *1/1 genotype; lane 3, 5: *1/2 genotypes; and lane 1: *2/2 genotype. (B) The mRNA levels of LAPTM4B in breast cancer tissues. The data represent one of three independent experiments.

In order to confirm the above results, we tested the genotypes and mRNA levels of LAPTM4B in 10 cases of breast cancer tissues with different genotypes of LAPTM4B (5 cases of *1/1, 4 cases of *1/2, 1 case of *2/2). As shown in Figure 3, the average mRNA levels of LAPTM4B/GAPDH were 5.73±0.80 in *1/2 genotype, 6.02±0.89 in *2/2 genotype and 1.00±0.13 in *1/1 genotype. No significant difference was found between *1/2 genotype to *2/2 genotype (p = 0.6999). The LAPTM4B*1/2 genotype and *2/2 genotype had higher levels of LAPTM4B compared with LAPTM4B*1/1 genotype (p = 0.0005 and P = 0.006 respectively). Therefore, allele *2 was also associated with relative higher level of LAPTM4B expression in breast cancer tissues.

### LAPTM4B Polymorphism and Breast Cancer Susceptibility

To investigate whether LAPTM4B polymorphisms is a susceptible biomarker of breast cancer, two hundred eleven healthy women and two hundred eight breast cancer patients were involved in this study. The average age of cancer cases and controls are 49.98±10.84 and 46.89±8.59 years respectively, and the Student’s t test showed no significant difference between the two groups (p = 0.297). We didn’t find statistically difference between the breast cancer patients and healthy controls in the matching characteristic. Chi-squared test was used to determine whether the subjects met the Hardy-Weinberg equilibrium. We confirmed that both case and control groups were in agreement with expectation on the basis of the Hardy–Weinberg equilibrium, for in case and control groups the χ2 value was 1.69 and 1.75 respectively, both p>0.05. These indicated that the selected samples represent the whole population.

In this case-control study, we found higher proportions of genotype *1/2 and *2/2 in breast cancer patients (48.08% and 8.65%, respectively) compared with healthy controls (36.02% and 2.84%, respectively). Crosstabs test indicates statistical significance in distribution of genotype*1/2 and*2/2 between the two groups. Odds ratio analysis showed that LAPTM4B*1/2, *2/2 and LAPTM4B (*1/2+*2/2) were associated with a significant increased risk of breast cancer compared with LAPTM4B*1/1 (OR = 1.328, 95% CI = 1.106–1.595; OR = 1.147, 95% CI = 1.046–1.257; and OR = 1.413, 95% CI = 1.169–1.707, respectively). It is also found that the frequency of allele *2 was higher in cases (32.69%) than in controls (20.85%). Patients carrying LAPTM4B*2 allele indicated 1.176-fold higher risk of developing breast cancer than those carrying LAPTM4B*1 (P<0.001, OR = 1.176, 95%CI = 1.082–1.278) (Table 1). Our findings suggested that LAPTM4B*2 was a risk factor for the development of breast cancer.

**Table 1:** Distribution of LAPTM4B alleles in cancer cases and controls.

	Number (%)		P value	OR (95%CI)
	Control (n = 211)	Cancer (n = 208)		
**Genotypes**				
*1/1	129(61.64%)	90(43.27%)		
*1/2	76(36.02%)	100(48.08%)	0.002	1.328(1.106–1.595)
*2/2	6(2.84%)	18(8.65%)	0.002	1.147(1.046–1.257)
*1/2+*2/2	82(38.86%)	118(56.73%)	<0.001	1.413(1.169–1.707)
**Alleles**				
*1	334(79.15%)	280(67.31%)		
*2	88(20.85%)	136(32.69%)	<0.001	1.176(1.082–1.278)

LAPTM4B, lysosome-associated protein transmembrane 4 beta; OR, odds ratio;

CI, confidence interval.

### LAPTM4B Polymorphism and Clinicopathological Variables

Table 2 summarized the association of LAPTM4B genotypes with clinicopathological characteristics, including age at diagnosis, pathological type, tumor size and differentiation classification, nodal metastasis and status of the estrogen and progesterone receptor status. We found that LAPTM4B (*1/2+ *2/2) genotypes are significantly associated with histopathologic grade (p = 0.0027) and lymph node metastasis (p = 0.0322) in breast cancer patients, but not with age, pathological type, tumor size, ER status, PR status, or C-erbB2 status.

**Table 2:** LAPTM4B genotype and clinicopathological variables.

Variables	LAPTM4B genotype		P value
	*1/1 *1/2+*2/2		
**Age**			0.6856
≤40	35	42	
>40	54	76	
**Pathological type**			0.1713
Ductal	75	95	
others	9	22	
**Tumor size(cm)**			0.3872
≤2	51	75	
>2	39	43	
**Histological grade**			**0.0027**
Low	74	72	
High	9	31	
**Lymph node metastasis**			**0.0322**
No	62	61	
Yes	21	43	
**ER status**			0.7579
Negative	40	9	
Positive	59	36	
**PR status**			0.179
Negative	29	37	
Positive	51	75	
**C-erbB2 status**			0.549
Negative	56	82	
Positive	24	30	
**Triple-negative status**			0.3844
No	74	90	
Yes	16	28	

LAPTM4B, lysosome-associated protein transmembrane 4 beta; ER, estrogen receptor;

PR, progestogen receptor; Triple-negative, ER, PR and C-erbB2 negative.

### LAPTM4B Polymorphism and Breast Cancer Prognosis

**Figure 4:**
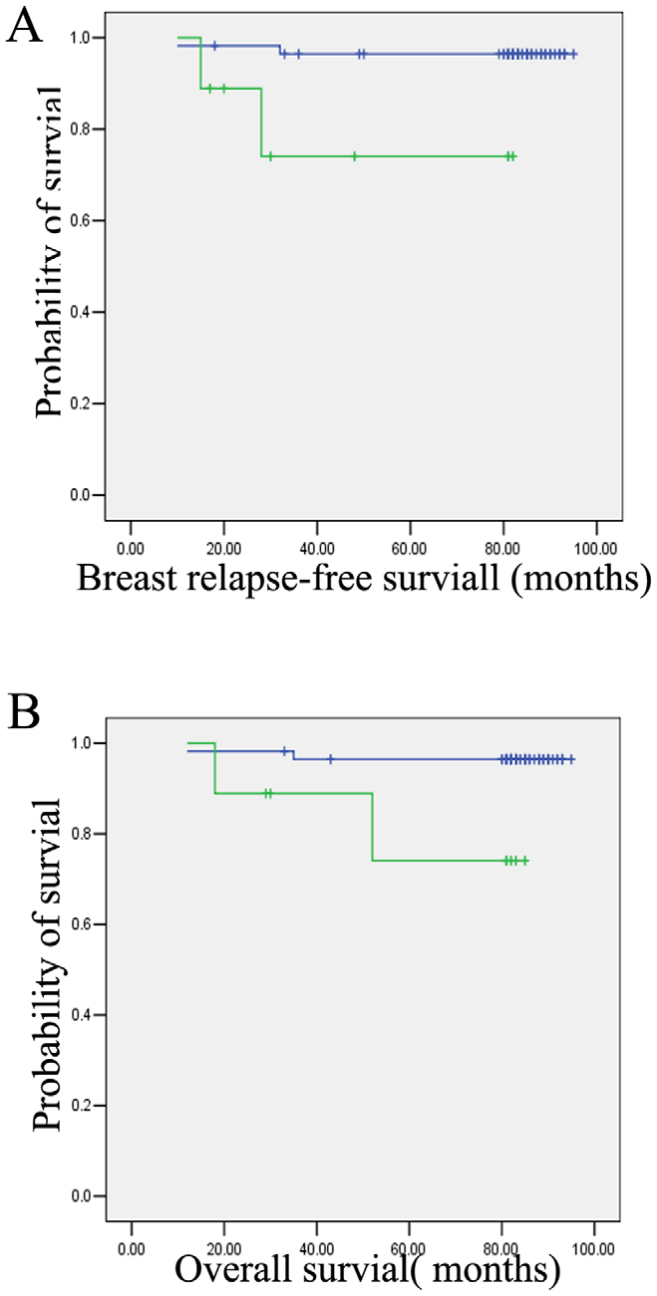
LAPTM4B genotypes and breast cancer prognosis. (A) genotype *2 of LAPTM4B had shorter breast relapse-free survival (P = 0.012). (B) Genotype *2 of LAPTM4B was associated with shorter overall survival (P = 0.018).

To evaluate the prognostic power of LAPTM4B polymorphism in breast cancer, we evaluated LAPTM4B genotype with respect to breast relapse-free survival and overall survival. As shown in Figure 4, genotype *2 of the LAPTM4B gene showed correlation both with shorter breast relapse-free survival and shorter overall survival in the sisty-six patients (P = 0.012, P = 0.018 respectively). We further performed Cox proportional hazards regression analysis of LAPTM4B polymorphism with the tissue samples stratified by other common clinicopathological parameters including age, ER, PR, HER-2, lymph node status and the sizes of primary tumors at the time of cancer diagnosis. LAPTM4B polymorphism failed to retain its prognostic significance in these analyses, which suggested that it was not a prognostic factor independent of other clinicopathological factors (P = 0.843).

## Discussion

Breast cancer is a major health problem and the leading cause of cancer deaths for women in the world. The tumorigenesis and progression of breast cancer are multistep processes accompanied by changes in host gene-expression patterns including tumor-suppressor genes, oncogenes and, simultaneously, by building up of the microenvironment [16], [17]. Therefore, it is important to find risks factors associated with breast cancer.

Previous studies had shown that the LAPTM4B gene was amplified in breast cancers, and demonstrated that amplification of LAPTM4B was associated with poor tumor response to anthracycline treatment and recurrence in women with primary breast cancer [18]. LAPTM4B mRNA and/or protein was also overexpressed in a wide variety of cancers such as hepatocellular carcinoma, gallbladder carcinoma, uterine cancer, ovarian cancer, extrahepatic cholangiocarcinoma [4], [19], [20], [21]. The overexpression of LAPTM4B was proved to be a novel molecular maker of progression, invasiveness and poor prognosis in various cancers [20], [22], [23], [24], [25], [26]. Overexpression of LAPTM4B could activate the AKT signaling pathway, upregulate the proliferation-promoting transcription factors such as c-myc, c-jun, and c-fos, and cell cycle-promoting proteins such as cyclin D1 and cyclin E to promote malignant transformation and enhance growth and metastasis [27], [28]. In addition, knockdown of LAPTM4B or mutation of the PPRP motif in the N-terminal region of LAPTM4B can attenuate its role in tumorigenesis and metastasis [28]. Therefore, LAPTM4B may also be a potential target for breast cancer therapy.

In this study, we first investigated the LAPTM4B genotypes in breast cancer cell lines and breast cancer tissues. By PCR and qRT-PCR analysis, we revealed LAPTM4B*2 allele had higher level of LAPTM4B expression compared with LAPTM4B*1 allele both in breast cancer cell lines and tissues. This proposed that LAPTM4B*2 allele may play a vital role in the development of breast cancer.

To further prove the role of LAPTM4B*2 in breast cancer, we investigated the LAPTM4B genotypes with PCR assays in 208 breast cancer patients and 211 healthy controls. We found that the allelic frequencies of the LAPTM4B*2 allele were 32.69% in the breast cancer group and 20.85% in the control group, representing a significant difference between these two groups. Our results were consistent with the findings reported by Fan et al. [29] and further confirmed that the LAPTM4B*2 allele was associated with significantly increased risk of breast cancer. By investigating the relationship between LAPTM4B genotype and clinicopathological variables of breast cancers, we found a strong association between LAPTM4B genotypes and histopathologic grade and lymph node metastasis in breast cancer patients. In breast cancer patients, genotype *2 of the LAPTM4B gene had both shorter disease-free survival and shorter overall survival by the Kaplan-Meier method. Though the genotypes were not an independent prognostic marker for overall survival of patients with breast cancer, this is needed to be further proved in larger number of cohorts.

In conclusion, our data demonstrated that allele LAPTM4B*2 was associated with breast cancer susceptibility. To the best of our knowledge, this is the first study to demonstrate the polymorphism of LAPTM4B was also associated with breast cancer progression and prognosis. These findings suggest that LAPTM4B*2 is a potential predicative marker for the susceptibility and prognosis of breast cancer. However the exact molecular mechanisms underlying the role of this polymorphism have not been worked out and further investigation of LAPTM4B*2 in breast cancer is warranted.
